# Ultrafast Transient Spectroscopy of Polymer/Fullerene Blends for Organic Photovoltaic Applications

**DOI:** 10.3390/ma6030897

**Published:** 2013-03-06

**Authors:** Sanjeev Singh, Zeev Valy Vardeny

**Affiliations:** Department of Physics & Astronomy, University of Utah, Salt Lake City, UT 84112, USA; E-Mail: sanjeev.729@gmail.com

**Keywords:** transient spectroscopy, organic photovoltaic, donor-acceptor blends, charge-transfer excitons

## Abstract

We measured the picoseconds (ps) transient dynamics of photoexcitations in blends of regio-regular poly(3-hexyl-thiophene) (RR-P3HT) (donors-D) and fullerene (PCBM) (acceptor-A) in an unprecedented broad spectral range of 0.25 to 2.5 eV. In D-A blends with maximum domain separation, such as RR-P3HT/PCBM, with (1.2:1) weight ratio having solar cell power conversion efficiency of ~4%, we found that although the intrachain excitons in the polymer domains decay within ~10 ps, no charge polarons are generated at their expense up to ~1 ns. Instead, there is a build-up of charge-transfer (CT) excitons at the D-A interfaces having the same kinetics as the exciton decay. The CT excitons dissociate into separate polarons in the D and A domains at a later time (>1 ns). This “two-step” charge photogeneration process may be typical in organic bulk heterojunction cells. We also report the effect of adding spin 1/2 radicals, Galvinoxyl on the ultrafast photoexcitation dynamics in annealed films of RR-P3HT/PCBM blend. The addition of Galvinoxyl radicals to the blend reduces the geminate recombination rate of photogenerated CT excitons. In addition, the photoexcitation dynamics in a new D-A blend of RR-P3HT/Indene C60 trisadduct (ICTA) has been studied and compared with the dynamics in RR-P3HT/PCBM.

## 1. Introduction

**Figure 1:**
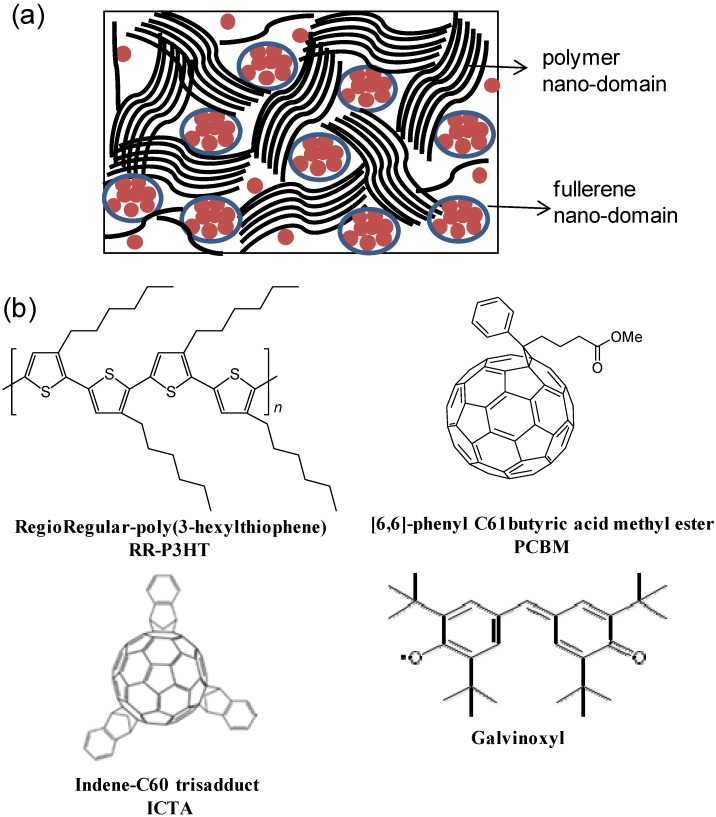
(color on line) (**a**) Schematic diagram of nano-domains of polymer (donor) and fullerene (acceptor) depicted in bulk heterojunction organic photovoltaic blend; (**b**) Schematic molecular structures of the Regio Regular (RR)-P3HT polymer, 6,6-phenyl-C_61_-butyric acid methyl ester (PCBM), Indene C60 trisadduct (ICTA) and Galvinoxyl radical.

In recent years, organic solar cells have attracted widespread interest in research institutes and commercial companies because of their versatility, flexibility, ease of processing and low cost. These factors contribute to the promise that organic solar cells may be able to provide a cost effective alternative compared to inorganic solar cells. In fact the most efficient organic photovoltaic (OPV) cells to date are based on bulk heterojunctions (BHJ) comprised of a blend of a π-conjugated polymer (PCP) and fullerene, having certified power conversion efficiency (PCE) around 9% [1,2,3]. The two organic semiconductors in the blend, dubbed donor (D-) and acceptor (A-), respectively, are cast from solution mixtures to form thin films with nanoscale domains of relatively pristine materials and large donor-acceptor (D-A) interface area. Formation of these nano domains is the key parameter to achieve efficient organic photovoltaic in BHJ solar cell. A schematic diagram of the blend with separate D- and A- nano-domains is shown in Figure 1a. In addition it has been shown that there also exist some amorphous regions in the blend where polymer and fullerene are finely mixed [4,5]. Despite these significant advances in device performance, the intricacies of charge carrier photogeneration and evolution in D-A blends for OPV applications are still the focus of fundamental research, with the goal of developing novel photoactive materials and improving the power conversion efficiency of solar cell devices [6,7].

The process of charge photogeneration in polymer/fullerene blend is usually described in a series of sequential steps as follows [6,7,8,9]: Absorption of an above-gap photon in the polymer domains generates intrachain exciton in the polymer chains. The exciton subsequently diffuses to the polymer/fullerene interface, where it may be quenched by electron transfer from the polymer to the fullerene. However the significant Coulomb attraction and/or wavefunction overlap between the electron-polaron in the fullerene domain and hole-polaron on the polymer domain results in the formation of charge transfer (CT) exciton state. Free charge carrier generation requires further dissociation of these initially generated CT excitons.

The role of the charge transfer states in charge photogeneration process has been hotly debated in the literature [6,7,8,9,10,11,12,13,14,15,16,17,18,19,20,21,22,23,24]. Initially, it was proposed that the photogenerated “donor exciton” diffusion to the interface is followed by ultrafast dissociation of the exciton without any intermediate state [25]. Later on, the CT state was confirmed by different spectroscopic techniques such as photothermal deflection spectroscopy [26], electroabsorption spectroscopy [27], photo- and electroluminescence measurements [28]. Importantly these CT exciton can be directly generated using below gap photoexcitation, without involving the first process of intrachain exciton generation in the donor domains [8,9,27,29,30,31]. With below-gap excitation the CT exciton may still dissociate into polarons in the polymer and fullerene domains. Drori *et al.* found that the photogenerated polarons with below-gap excitation are trapped at the interfaces with relatively long lifetime compared to polaron photogenerated with above gap excitation [31]. The interaction of donor and acceptor molecules creates a series of charge transfer state. In a separate three beam measurement, Bauklin *et al.* studied in detail the higher energy level (hot) CT states in a series of donor and acceptor materials, and found that these states are delocalized and help in charge separation [30]. On the other hand, Lee *et al.* studied photovoltaic blends using Fourier-transform photocurrent spectroscopy and photo-thermal deflection spectroscopy, and found that the internal quantum yield of carrier photogeneration is similar for both excitons and direct excitation of charge transfer states [15]. It is therefore clear that the properties and dynamics of photoexcitations in PCPs and PCP/fullerene blends are of fundamental importance because they play an essential role in the device operation.

This review paper summarizes the early stages of the charge photogeneration process in D-A blend of polymer regio-regular (RR-)(3 hexyl thiophene) (P3HT; see Figure 1b) and two fullerene acceptor molecules 6,6-phenyl-C_61_-butyric acid methyl ester (PCBM) and Indene C60 trisadduct (ICTA); Figure 1b using the pump/probe transient photomodulation spectroscopy in a broad spectral range. In addition, we have also studied the effect of a spin-1/2 radical “Galvinoxyl” on the charge photogeneration process in RR-P3HT/PCBM blend which ultimately affects the photovoltaic device performance of this blend [32,33].

The RR-P3HT/PCBM with (1.2:1) weight ratio blend shows high solar power conversion efficiency, η ~ 4% [8,31] because of phase separation of D-A domains. We show that after the photoexcited excitons in the polymer domains reach the D-A interfaces, the charge generation process proceeds via the formation of CT excitons at the interfaces, which occurs within ~10 ps. These findings are consistent with our recent report about ultrafast photophysics of RR-P3HT/Indene C_60_ bisadduct (ICBA) blend [9]. Finally, we studied a new fullerene acceptor molecule, namely Indene C_60_ trisadduct (ICTA) (Figure 1b) of which LUMO level is higher than that in PCBM, which ultimately yields larger open circuit voltage (~1.08 V) for RR-P3HT/ICTA blend compared to the value of 0.6 V for RR-P3HT/PCBM blend. Our findings support the two-step process of the charge photogeneration mechanism in organic D-A blends proposed before [9], and emphasize the important role of the CT exciton binding energy in generating free charges in organic solar cells.

## 2. Experimental

The P3HT polymers, PCBM and ICTA fullerenes were supplied by Plextronics Inc. The mixing ratio of the P3HT/PCBM blend and P3HT/ICTA blend was 1.2:1 by weight, which gives the optimal η-value in solar cells. The films were spin cast onto CaF_2_ or CsI that are transparent in the mid-IR spectral range. The RR-P3HT/PCBM and RR-P3HT/ICTA blend film was thermally annealed at 150 °C for 30 min to enhance the D-A domains size [34]. Galvinoxyl was purchased from Aldrich Corp., and added to the P3HT/PCBM blend in several weight percentages; here we focus on mixtures having the respective percentage of 3% and 10%.

For the polarized transient photomodulation (PM) spectroscopy we used the femtosecond two-color pump/probe correlation technique with two different laser systems based on a Ti: Sapphire oscillator [8,31]. These are: a low-power (energy/pulse ~0.1 nJ) high repetition rate (~80 MHz) laser system for the mid-IR spectral range; and a high power (energy/pulse ~10 µJ) low repetition rate (~1 kHz) laser system for the near-IR/visible spectral range. The pump excitation photon energy, *ħ*ω(*pump*) was set at 3.1 eV for above-gap and 1.55 eV for below-gap excitation, respectively. The pulse energy flux on the sample was adjusted so that the initial photoexcited exciton density, *N*(0) ≈ 10^16^/cm^3^ (*N*(0) ≈ 10^17^/cm^3^) for the mid-IR (near-IR) laser system. For the probe in the mid-IR measurements we used an optical parametric oscillator (OPAL, Spectra Physics) that generates *ħ*ω(*probe*) ranging from 0.55 to 1.05 eV. In addition, we also used a difference-frequency crystal (AgGaS_2_) and the “signal” and “idler” beams of the OPAL for generating *ħ*ω(*probe*) in the spectral interval 0.25 to 0.43 eV. The pump beam was modulated at frequency, *f* = 40 kHz and the changes, Δ*T* in the probe transmission, *T* were measured using a phase-sensitive technique. For the transient near-IR/visible spectroscopy measurements we used white light super-continuum as probe, spanning the spectral range from 1.15 to 2.5 eV; the pump modulation frequency here was synchronized with the laser rep. rate. The transient photomodulation signal, Δ*T*/*T* is positive for photoinduced absorption (PA) and negative for photo-bleaching and stimulated emission. Δ*T*(*t*)/*T* was measured using a computer controlled translation stage up to 2 ns, with time resolution of ~150 fs set by the pump/probe cross-correlation. Δ*T*(0)/*T* spectra from the two laser systems were normalized to each other at several wavelengths in the near-IR/visible spectral range [35].

For the mid-IR high repetition laser system it is possible that the time interval between two successive pulses may be longer than the recombination lifetime of some photoexcitations, and therefore a background (BG) PA may be formed in the probe transmission. The background PA is modulated at frequency of 40 kHz (coming from the pump beam modulation), and thus is sensitive to long-lived photoexcitations in the film having lifetime, τ < ~1/*f*. An advantage of the mid-IR laser system is thus that the background (BG) PA spectrum can be readily measured using the same pump/probe set up as for the ultrafast response. This was achieved by measuring the PA signal at delay time t < 0, since the probe pulse arrives before the pump pulse and therefore is affected by the “left-over” photoexcitations in the film that survive in between successive pulses [36].

### 2.1. Photoexcitations Dynamics in Pristine P3HT

**Figure 2:**
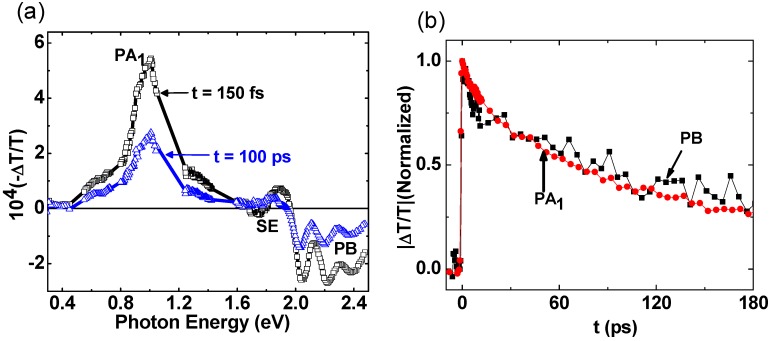
(color on line) (**a**) The transient photomodulation spectrum of pristine RR-P3HT film at *t* = 150 fs and *t* = 100 ps, respectively. The exciton bands PA_1_, SE and PB are indicated; (**b**) The transient decay of PA_1_ and PB bands up to t = 180 ps.

Since the majority of primary photoexcitations are created in the donor material, it is advisable to first analyze Δ*T*(*t*)/*T* spectra of *pristine* polymer. Figure 2a shows Δ*T*(*t*)/*T* spectra of pristine RR-P3HT polymer at two different time scales, namely *t* = 150 fs (within pulse duration) and *t* = 100 ps. Δ*T*(0)/*T* spectrum contains a single PA band, namely PA_1_ at 1 eV and photo-bleaching (PB) above 1.97 eV. In addition there is a small stimulated emission band, SE at 1.75 eV that disappears at *t* = 100 ps [8]. Figure 2b shows the decay of PA_1_ and PB in pristine RR-P3HT polymer. These two bands decay together, having an exponential time constant τ_0_ = 70 ps, and we thus conclude that they are due to photogenerated excitons. No photogenerated polarons with PA bands at 0.1 and 1.8 eV [8,37,38] are observed, and we thus conclude that the primary photoexcitations in high quality pristine polymer are singlet excitons.

### 2.2. Photoexcitations Dynamics in P3HT/PCBM Blend

**Figure 3:**
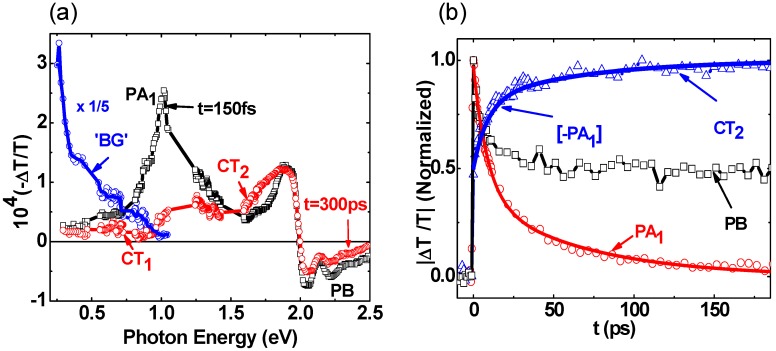
(color on line) (**a**) The transient photomodulation spectrum of RR-P3HT/PCBM blend film at *t* = 0 and *t* = 300 ps, respectively; the exciton band PA_1_, and CT exciton bands CT_1_ and CT_2_ are indicated. The blue circles and line represent the background (BG) PA spectrum measured at *t* = −5 ps; (**b**) The transient decay of PA_1_, build-up dynamics of CT_2_, and the PB decay up to 180 ps. The line through the data points is a fit using the Förester resonant energy transfer (FRET) mechanism (see text); the same function also fits the CT_2_ build-up dynamics.

Figure 3a shows a Δ*T*(*t*)/*T* spectra of RR-P3HT/PCBM blend. Δ*T*(0)/*T* spectrum is very similar to that of pristine RR-P3HT (Figure 2a), indicating that in the blend excitons are also initially photogenerated in the polymer domains. At *t* > 0 the excitons decay, however *no polarons are generated* at the expense of the exciton decay up to 300 ps, since there is no PA build-up at low *ħ*ω(probe) where the polaron P_1_ band dominates the absorption spectrum [8,37,38]. We thus conclude that the excitons in the polymer domains decay into a *new state* that is not due to separated free polarons. This new state must be related to the D-A interfaces in the films, and we thus propose that is a CT exciton at the D-A interface. In contrast, the background PA spectrum in the mid-IR (Figure 3a) is very similar to the P_1_ band in the polaron doping induced absorption spectrum [8,37,38], showing that charge polarons are indeed photogenerated in this RR-P3HT/PCBM film, in agreement with the high solar cells efficiency based on this blend [8]. We conclude that the charge photogeneration process in the blend proceeds in two stages [8,15]. The first stage is exciton trapping in CT states at the D-A interfaces, followed by a much slower exciton dissociation into free polarons in the D and A domains.

The exciton decay dynamics in the blend is much faster than in the pristine polymer (PA_1_ in Figure 2b). The shorter lifetime in the blend is related to the exciton dynamics towards the D-A interfaces. PA_1_ decay cannot be fit with a single or few exponential decay functions; nor can it be fit using a diffusion model [~ (1 + *t*/τ)^−1^]. Alternatively, PA_1_ decay can be fit using multiple power-law decays that originate from a Förester resonant energy transfer (FRET) into the CT exciton [39], averaged over the exciton initial distance from the D-A interface. A detailed study of PA_1_ decay kinetics for the donor polymer in the RR-P3HT/PCBM blend system was recently published [8] This model yields the following time dependent surviving exciton density *N*(*t*) in the polymer domains;

*N*(*t*)/*N*(0) = exp(−*t*/τ_0_)[m_1_ + m_2_(C_1_*t*^1/2^ – C_2_*t*^1/3^ + C_3_*t*^1/6^)] (1)

where τ_0_ = 70 ps is the natural exciton lifetime in RR-P3HT that is obtained from the PA dynamics of Figure 2b; m_1_ and m_2_ are fitting parameters; and the C constants are given by the relations: C_1_ = 0.2 u^−3^, C_2_ = 0.66 u^−2^ and C_3_ = 0.54/u, where u = *D*/2*R*_0_ is the parameter ratio of the grain size, *D* to twice the FRET radius, *R*_0_, which was measured before to be between 3 and 9 nm [40]. Using *R*_0_ = 6 nm and *D*= 16 nm from the XRD studies [8], we obtain u = 1.3. Subsequently the excellent fit to the PA_1_ decay seen in Figure 3b was obtained using m_1_ = 0.14 and m_2_ = 7.

**Figure 4:**
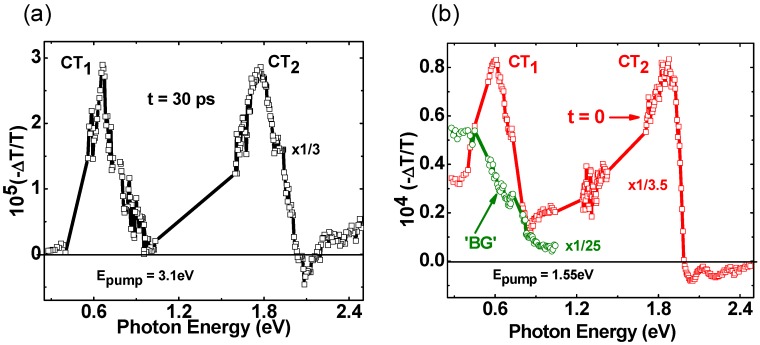
(color on line) (**a**) The transient photomodulation spectrum of RR-P3HT/PCBM blend film at *t* = 30 ps excited at 3.1 eV, normalized and subtracted from the spectrum at *t* = 0, that shows the two newly formed CT_1_ and CT_2_ bands; (**b**) Same as in (**a**) but at *t* = 0 and excited at 1.55 eV, which is below the gap of both polymer and fullerene constituents. The green circles and line represent the background (BG) PA spectrum measured at t = −5 ps.

In support of the CT intermediate role in the charge photogeneration process in the blend, Figure 3a also shows that PA build-up indeed occurs in both mid- and near-IR [11] spectral ranges. In fact there are two PA bands, namely CT_1_ in the mid-IR and CT_2_ in the near-IR that are generated at longer time *at the expense of the exciton PA_1_ decay*. Figure 3b shows that the CT_2_ build-up dynamics in the near-IR closely matches the exciton PA_1_ decay, since the same function of time fits both PA_1_ decay and CT_2_ build-up dynamics (measured at 1.75 eV probe). Figure 4a more clearly shows the two PA bands that are generated at the expense of the exciton PA_1_ decay. To obtain the CT spectrum we subtracted the photomodulation spectrum at *t* = 30 ps from that at *t* = 0, after normalizing the two PA bands at 1 eV and 2 eV for the CT_1_ and CT_2_ bands, respectively. It is seen that the CT spectrum contains two prominent PA bands that peak at 0.6 (CT_1_) and 1.75 eV (CT_2_), respectively, which are very different than the bands P_1_ and P_2_ of polarons [8]. Consequently we propose that these two PA bands are due to optical transitions within the CT manifold at the D-A interfaces [30].

To support this assumption we measured the transient photomodulation spectrum using *ħ*ω(pump) = 1.55 eV, which is below the optical gap of the polymer and fullerene constituents. Such low *ħ*ω(pump) can resonantly excite the CT state at the D-A interface, since its energy was measured to be between 1.2 to 1.6 eV [31], without photogenerating excitons in the polymer domains. Figure 4b shows that under these conditions the two CT PA bands are *instantaneously* photogenerated; which is compelling evidence that they originate from the CT states at the interfaces. This supports our assignment for the CT bands in the transient photomodulation spectrum of this blend.

Interestingly the background PA spectrum excited with below-gap pump excitation (Figure 4b) is very similar to that generated using above-gap pump excitation (Figure 3a), which we identified as due to long-lived charge polarons. This shows that there exists a mechanism where thermalized CT excitons at the D-A interfaces are able to separate into free polarons in the donor and acceptor domains, *regardless of the initial ħ*ω(pump) [30]. This finding is very important, since it can refute the notion that the CT state in the blend lies too deep in the gap to have any influence over the charge photogeneration process. Apparently the exciton kinetic energy when reaching the CT state plays a minor role in the charge photogeneration process; this may explain the flat spectral response of the photocurrent action spectrum in organic solar cells [31].

### 2.3. Photoexcitation Dynamics in P3HT/PCBM Blend Doped with Galvinoxyl Spin 1/2 Radicals

**Figure 5:**
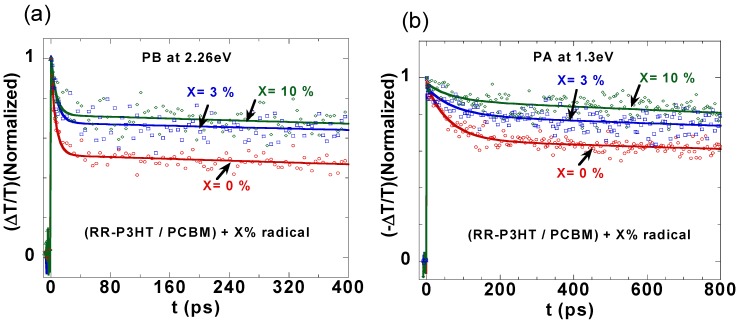
(color on line) The transient decays in RR-P3HT/PCBM (1.2:1) films mixed with 0%, 3% and 10% of Galvinoxyl impurities measured at: (**a**) 2.26 eV (PB) and (**b**) 1.3 eV (CT_2_).

We have found that the addition of few weight percentage of a spin-1/2 radical “Galvinoxyl” to the RR-P3HT/PCBM blend improves the photovoltaic device performance of the solar cell based on this blend. This occurs since all three OPV components including the short-circuit current density, *J_sc_*, fill-factor, and power conversion efficiency dramatically increase [32,33]. In order to explore the origin of this OPV improvement, we studied the photoexcitation dynamics of RR-P3HT/PCBM blend “doped” with three different weight percentages (0%, 3% and 10%) of Galvinoxyl spin-1/2 radicals. First,we studied the decay of the polymer photobleaching (PB) band at 2.26 eV for different radical-doped blend films at 0%, 3% and 10% radical impurities, as shown in Figure 5a. The PB decay is directly related to the ground state recovery of the excited species in the *P3HT polymer*, since PCBM does not strongly absorb in this spectral range, and the radical concentration is relatively small. We therefore conclude that the PB decay here is related to bound polaron recombination in the P3HT polymer. We note that the decay rate *decreases* upon adding the radical impurities to the blend. We fitted these decay dynamics using a double exponential decay function that contains a short (~7 ps) and long (>5 ns) lifetime components. The best-fit lifetimes and relative contributions of the double exponentials are given in Table 1. The PB decay dynamics indicates that the addition of radical impurities to the D-A mixture *reduces the contribution of short time recombination losses in the blend*.

**Table 1:** The transient decay parameters (lifetime and relative contribution) and photovoltaic device parameters of annealed polymer regio-regular 3 hexyl thiophene/6,6-phenyl-C_61_-butyric acid methyl ester PCBM (RR-P3HT/PCBM) (1.2:1) films with different percent (%) of Galvinoxyl radical impurities.

Galvinoxyl (%)	Transient Decay at 2.26 eV	Transient Decay at 1.3 eV	*J*_sc_ (mA/cm^2^)	*V*_oc_ (V)	Fill-Factor (%)	Efficiency (%)
0	7 ps (47%), >5 ns (53%)	62 ps (32%), >5 ns (68%)	11.3	0.57	55	3.46
3	8 ps (33%), >5 ns (67%)	73 ps (16%), >5 ns (84%)	12.7	0.58	60	4.20
10	7 ps (28%), >5 ns (72%)	63 ps (10%), >5 ns (90%)	11.2	0.61	53	3.55

Figure 5b shows the decay of CT_2_ band at 1.3 eV for the various radical-doped blend films, as in Figure 5a above. The CT_2_ band is associated with excited state absorption from the bound polaron species in the polymer chains, and consequently its decay dynamics show geminate (fast) recombination. From transient measurements of pure PCBM film we verified that polarons in PCBM film do not have strong PA in this spectral range. CT_2_ decay dynamics are again fitted with a double exponential function as given in Table 1. Similar to the PB band dynamics, CT_2_ decay also contains a short (~68 ps) and long (>5 ns) lifetime components, and their contributions changes with % of radical added. The important discovery here is that *the addition of radical impurities to the blend reduces the geminate (fast) recombination*. We note, however that the short decay component of PB (~7 ps) does not match with the short decay component of CT_2_ (~68 ps) because PB has contributions from *all* the excited state species, including singlet excitons whose PA bands do not match the 1.3 eV probe photon-energy.

The effect of Galvinoxyl radical on the rate of geminate recombination can be explained as following. Galvinoxyl is a spin 1/2 radical, and this opens up the possibility of spin-spin interaction with the photogenerated species in the blend. In particular we speculate that the radical spin may interact with the electron and hole spins that are in the “singlet” configuration in the photogenerated CT species that exist at the interface of P3HT and PCBM. This spin-spin interaction may cause a spin flip of one of the locked spins in the CT singlet configuration. Consequently the bound CT may undergo a fast intersystem crossing into the spin-triplet configuration that traditionally is longer-lived. The triplet CT species would therefore be more susceptible for efficient dissociation at a later time into free electron- and hole-polarons in the PCBM and P3HT polymer phases, respectively; and this increases the photogenerated current density in the organic photovoltaic (OPV) devices made of these radical/blend mixtures.

To see the effect of reduction in geminate recombination rate of polarons due to the spin 1/2 radical impurities in OPV devices, we fabricated OPV devices based on radical/blend mixtures films, with various radical percentages as active layer. The device characteristic parameters such as short-circuit current density, *J*_sc_, fill-factor, open circuit voltage and power conversion efficiency are given in Table 2. As seen, the devices show increase in all typical parameters at ~3% addition of radical, especially in *J*_sc_, which is consistent with the reduction in geminate recombination rate that we found by the ultrafast spectroscopy here. These parameters, however, start decreasing at 10% radicals, which might be due to change in morphology of the blend film at higher percentage of radicals that should decrease carrier mobility. The reduction in *J*_sc_ and FF of solar cells with 10% radicals supports this conclusion.

**Table 2:** The transient decay parameters (lifetime and relative contribution) of annealed RR-P3HT/PCBM and RR-P3HT/ICTA (Indene C60 trisadduct) (1.2:1) films.

Sample	Transient Decay at 2.26 eV	Transient Decay at 1.3 eV
RR-P3HT/PCBM	7 ps (47%), >5 ns (53%)	60 ps (32%), >5 ns (68%)
RR-P3HT/ICTA	9 ps (57%), ~2.5 ns (43%)	34 ps (36%), ~3.3 ns (64%)

**Figure 6:**
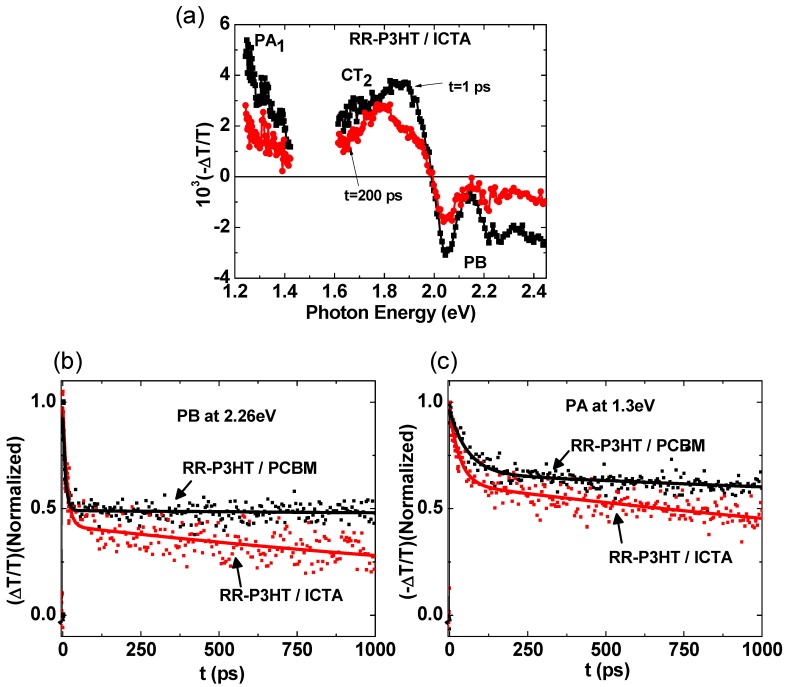
(color on line) (**a**) The transient photomodulation spectrum of RR-P3HT/ICTA blend at *t* = 1 ps and *t* = 200 ps, respectively excited at 3.1 eV; the PA bands PB, PA_1_ and CT_2_ are assigned; (**b**) The transient decay of PB (2.26 eV) and the decay of CT_2_ (1.3 eV).

### 2.4. Photoexcitation Dynamics in P3HT/ICTA Blend

We have also studied the charge photogeneration of RR-P3HT/ICTA (1.2:1). Figure 6a shows Δ*T*(*t*)/*T* spectra of RR-P3HT/ICTA in the visible near-IR wavelength range at two different time scales, namely *t* = 1 ps and *t* = 200 ps. Δ*T*(1 ps)/*T* spectrum contains PB above 1.97 eV and CT_2_ in the range of 1.8 eV. The photovoltaic devices based on RR-P3HT/ICTA shows poor power conversion efficiency of 0.6% compared to 4% for the RR-P3HT/PCBM. The origin of this difference can be seen in the decay of RR-P3HT/ICTA blend at 2.26 eV (PB) and 1.3 eV (CT_2_). Figure 6b,c shows the comparison of the decays of these two blends at 2.26 eV (PB) and 1.3 eV (CT_2_), respectively up to 1 ns. It is clear that the decay of PB and CT_2_ are faster in the blend of RR-P3HT/ICTA compared to those in RR-P3HT/PCBM. The decay kinetics was fitted with bi-exponential function, and the fitting parameters are shown in the Table 2.

## 3. Conclusions

In summary, using the ps pump/probe photomodulation technique in P3HT/PCBM blends with typical D-A bulk heterojunction morphology, we demonstrated that the charge photogeneration mechanism in organic solar cells occurs in two-steps. First, the photogenerated excitons in the polymer domains reach the D-A interfaces within a few ps depending on the domain size, where they form CT excitons. This process is followed by CT exciton dissociation into free charged polarons in the D and A domains in the ns time scale. We have also shown the addition of spin 1/2 radical reduces the geminate recombination of RR-P3HT/PCBM blend. In addition, the blend of RR-P3HT/ICTA shows faster recombination compared to the blend of RR-P3HT/PCBM.
